# Effects of non-pharmaceutical interventions on COVID-19 cases, deaths, and demand for hospital services in the UK: a modelling study

**DOI:** 10.1016/S2468-2667(20)30133-X

**Published:** 2020-06-02

**Authors:** Nicholas G Davies, Adam J Kucharski, Rosalind M Eggo, Amy Gimma, W John Edmunds, Centre for the Mathematical Modelling of Infectious Diseases COVID-19 working group, Thibaut Jombart, Kathleen O'Reilly, Akira Endo, Joel Hellewell, Emily S Nightingale, Billy J Quilty, Christopher I Jarvis, Timothy W Russell, Petra Klepac, Nikos I Bosse, Sebastian Funk, Sam Abbott, Graham F Medley, Hamish Gibbs, Carl A B Pearson, Stefan Flasche, Mark Jit, Samuel Clifford, Kiesha Prem, Charlie Diamond, Jon Emery, Arminder K Deol, Simon R Procter, Kevin van Zandvoort, Yueqian Fiona Sun, James D Munday, Alicia Rosello, Megan Auzenbergs, Gwen Knight, Rein M G J Houben, Yang Liu

**Affiliations:** a Department of Infectious Disease Epidemiology, London School of Hygiene & Tropical Medicine, London, UK

## Abstract

**Background:**

Non-pharmaceutical interventions have been implemented to reduce transmission of severe acute respiratory syndrome coronavirus 2 (SARS-CoV-2) in the UK. Projecting the size of an unmitigated epidemic and the potential effect of different control measures has been crucial to support evidence-based policy making during the early stages of the epidemic. This study assesses the potential impact of different control measures for mitigating the burden of COVID-19 in the UK.

**Methods:**

We used a stochastic age-structured transmission model to explore a range of intervention scenarios, tracking 66·4 million people aggregated to 186 county-level administrative units in England, Wales, Scotland, and Northern Ireland. The four base interventions modelled were school closures, physical distancing, shielding of people aged 70 years or older, and self-isolation of symptomatic cases. We also modelled the combination of these interventions, as well as a programme of intensive interventions with phased lockdown-type restrictions that substantially limited contacts outside of the home for repeated periods. We simulated different triggers for the introduction of interventions, and estimated the impact of varying adherence to interventions across counties. For each scenario, we projected estimated new cases over time, patients requiring inpatient and critical care (ie, admission to the intensive care units [ICU]) treatment, and deaths, and compared the effect of each intervention on the basic reproduction number, *R*_0_.

**Findings:**

We projected a median unmitigated burden of 23 million (95% prediction interval 13–30) clinical cases and 350 000 deaths (170 000–480 000) due to COVID-19 in the UK by December, 2021. We found that the four base interventions were each likely to decrease *R*_0_, but not sufficiently to prevent ICU demand from exceeding health service capacity. The combined intervention was more effective at reducing *R*_0_, but only lockdown periods were sufficient to bring *R*_0_ near or below 1; the most stringent lockdown scenario resulted in a projected 120 000 cases (46 000–700 000) and 50 000 deaths (9300–160 000). Intensive interventions with lockdown periods would need to be in place for a large proportion of the coming year to prevent health-care demand exceeding availability.

**Interpretation:**

The characteristics of SARS-CoV-2 mean that extreme measures are probably required to bring the epidemic under control and to prevent very large numbers of deaths and an excess of demand on hospital beds, especially those in ICUs.

**Funding:**

Medical Research Council.

## Introduction

Severe acute respiratory syndrome coronavirus 2 (SARS-CoV-2) has spread to multiple countries after causing an initial outbreak of COVID-19 in Wuhan, China.^1^ Early evidence indicated SARS-CoV-2 was capable of sustained human-to-human transmission^2^ and could cause severe disease,^3^ with a higher risk of severe and fatal outcomes in older individuals.^4^ The first two cases of COVID-19 in the UK were confirmed on Jan 31, 2020. Although implementation of testing, isolation, and contact tracing probably slowed early transmission,^5^ it was not sufficient to contain the outbreak in the UK.

Following the introduction of extensive control measures in Wuhan in late January, including—among other measures—travel restrictions, physical distancing, and requirements for residents to stay within their homes, there was a substantial decline in local transmission.^6^, ^7^, ^8^ Physical distancing measures, such as closure of schools, retail businesses, and restaurants, as well as constraints on individual movements and social interactions, are now in place in many countries with the aim of reducing transmission of SARS-CoV-2.^9^, ^10^

Several studies have explored the potential effect of control measures on the dynamics of COVID-19.^8^, ^11^, ^12^, ^13^, ^14^, ^15^ These studies have broadly suggested that moderate measures could reduce epidemic size, but more intensive measures would be required to ensure health system capacity was not surpassed.

**Research in context**

**Evidence before this study**

As countries have moved from early containment efforts to planning for the introduction of large-scale non-pharmaceutical interventions to control COVID-19 outbreaks, epidemic modelling studies have explored the potential for extensive physical distancing measures to curb transmission. However, it remains unclear how different combinations of interventions, timings, and triggers for the introduction and lifting of control measures could affect the impact of the epidemic on health services, and what the range of uncertainty associated with these estimates would be.

**Added value of this study**

Using a stochastic, age-structured epidemic model, we explored how eight different intervention scenarios could influence the number of new cases and deaths, as well as intensive care beds required over the projected course of the epidemic. We also assessed the potential impact of local versus national targeting of interventions, reduction in leisure events, increased childcare by grandparents, and timing of triggers for different control measures. We simulated multiple realisations for each scenario to reflect uncertainty in possible epidemic trajectories.

**Implications of all the available evidence**

Our results support early modelling findings, and subsequent empirical observations, that in the absence of control measures, a COVID-19 epidemic could quickly overwhelm a health-care system. We found that even a combination of moderate interventions—such as school closures, shielding of older people, and self-isolation of symptomatic individuals—would be unlikely to prevent an epidemic that would far exceed available intensive care unit capacity in the UK. Intermittent periods of more intensive lockdown-type measures are predicted to be effective for preventing the health-care system from being overwhelmed.

However, it remains unclear precisely how the timing, duration, and intensity of different measures targeting transmission and burden can reduce the impact of COVID-19 in the UK. Here, based upon scenarios originally presented to scientific advisory bodies in the UK, we use a mathematical model to assess the potential impact of different control measures for mitigating the burden of COVID-19 in the general UK population, and evaluate possible medium-term scenarios as the most restrictive short-term measures are eventually lifted.

## Methods

### Dynamic transmission model

**Figure 1:**
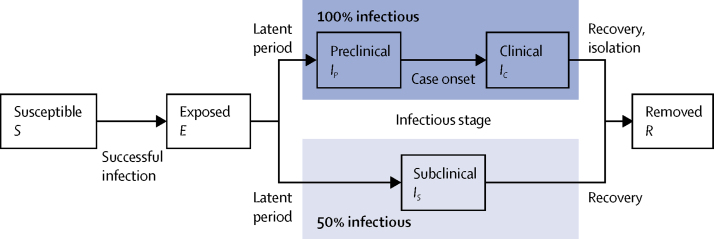
State transitions in the model Individuals in the stochastic compartmental model are classified into susceptible, exposed, infectious (preclinical, clinical, or subclinical), and recovered states (ie, removed from the model). The model is stratified into 5-year age bands and epidemics are simulated in the 186 county-level administrative units of the UK.

In this modelling study, we analysed a stochastic compartmental model stratified into 5-year age bands, with individuals classified according to current disease status (figure 1) and transmission between age groups in the community based on UK social mixing patterns (full details in the appendix pp 2–5).^16^, ^17^ Briefly, all individuals in the model start as susceptible to SARS-CoV-2 infection, and enter the exposed state upon effective contact with an infectious person. After a latent period lasting 4 days on average, exposed individuals become infectious, either with a preclinical infection (lasting 1·5 days on average) followed by a clinical infection (lasting 3·5 days on average), or with a subclinical infection (lasting 5 days on average). After the infectious period, individuals enter the removed state due to recovery or isolation and cannot be reinfected. Subclinical infections are those which result in few or no symptoms and hence, along with preclinical infections before symptom onset, are unlikely to be ascertained under syndromic surveillance; we assumed that subclinical infections are 50% as infectious as preclinical and clinical infections. COVID-19 shows markedly different dynamics in children than in adults.^16^ Therefore, we assumed that older individuals are more likely to show clinical symptoms, adopting the results of an analysis of COVID-19 case data across six countries from December, 2019, to March, 2020 (appendix p 4).^16^ The model tracks 66·4 million people aggregated to the 186 county-level administrative units in England, Wales, Scotland, and Northern Ireland.

We ran 200 stochastic simulations for each modelled scenario, choosing a randomly selected value for the basic reproduction number *R*_0_—which describes the average number of secondary infections caused by a typical primary infection in a completely susceptible population—for each simulation; the random values of *R*_0_ were chosen from a normal distribution with mean 2·7 (SD 0·6; see section on key model parameters below). We also chose a random date of SARS-CoV-2 introduction for each administrative unit, with London boroughs seeded within the first week and all other administrative units seeded within the first 4 weeks of the epidemic (appendix pp 3–4). Case estimates are of clinical cases (figure 1). For each scenario, we report the median outcome and 95% prediction intervals (PIs), which are generated from the 2·5th and 97·5th quantiles of the resulting distribution of simulation results. The model was implemented in R (version 3.6.3) and C++. An independent CODECHECK analysis has verified the model results are reproducible.^18^

### Key model parameters

We collated multiple sources of evidence to estimate key model parameters (appendix p 10). In a meta-analysis of studies and preprints published before Feb 26, 2020, we estimated that *R*_0_ was 2·7 (95% credible interval 1·6–3·9) across settings without substantial control measures in place (appendix pp 3–4). We used age-stratified case-fatality ratios (CFRs) estimated using case data from China up to Feb 11, 2020;^4^, ^19^ these CFRs ranged substantially across age groups from 0·1% in people aged 20–29 years to 7·7% in those aged 80 years or older. These CFRs were assumed to capture the risk of death from COVID-19 independently of hospitalisation status. Using these values along with the scaling between CFR and hospitalised cases, we also estimated the proportion of clinical cases in each age group that would require hospitalisation, which was 0·8% in the 20–29-year age group and 62% in those aged 80 years or older (appendix p 11). All differences between children and adults in the model are captured by age-specific contact rates, the age-specific probability of developing clinical symptoms of COVID-19 upon infection by SARS-CoV-2, and age-specific differences in the rate of hospitalisation and the CFR.

### Intervention scenarios

The non-pharmaceutical interventions we analysed were school closures, physical distancing, shielding of older people (ie, ≥70 years), self-isolation of symptomatic individuals, and a combination of all four policies. We assumed these interventions would affect the rate of contact between individuals, as well as the relative infectiousness of clinically infected individuals (but not preclinically or subclinically infected individuals) in the case of self-isolation of symptomatic individuals. Separate contact matrices were constructed for contacts made at home, at work, at school, and in other contexts (ie, leisure, transport, and in other places), calculated from survey data collected in Great Britain in 2006.^17^ Interventions were assumed to uniformly decrease the number of contacts between each pair of age groups in these matrices, altering the relative number of contacts of each type according to our best estimate of a reasonable reduction in contact rates under each scenario (table 1).

**Table 1:** Effect of intervention scenarios on contact rates and infectiousness of clinically infected individuals

	**Home contacts**	**Work contacts**	**School contacts**	**Other contacts**	**Infectiousness of clinically infected individuals**
Baseline	100%	100%	100%	100%	100%
School closures	100%	100%	0%	100%	100%
Physical distancing	100%	50%	100%	50%	100%
Shielding of older people	100%	25% (≥70 years); 100% (others)	100%	25% (≥70 years); 100% (others)	100%
Self-isolation	100%	100%	100%	100%	65%
Combined*	100%	25% (≥70 years); 50% (others)	0%	25% (≥70 years); 50% (others)	65%
Intensive interventions	100%	25% (≥70 years); 65% (others)	100% (schools open); 0% (schools closed)	16% (≥70 years); 59% (others)	65%
Lockdown	100%	10%	10% (schools open); 0% (schools closed)	10%	65%

Data are the percentages of contact rates or individual infectiousness remaining after each intervention; each intervention was assumed to affect either a component of the contact matrix or the infectiousness of clinically infected individuals, reducing it to the percentage shown. Where interventions include shielding of older people, percentages of contact rates are given separately for people aged 70 years or older and all other people.

* School closures, physical distancing, shielding of older people, and self-isolation combined.

Shielding of older people was simulated by reducing contacts only for rows and columns of the contact matrix corresponding to individuals aged 70 years or older, and the overall contact matrix was the sum of the home, work, school, and other matrices after interventions were applied. We simulated self-isolation of symptomatic individuals by decreasing their infectiousness by 35% during the intervention period. This was based on a calculation that approximately 70% of contacts occur outside the home;^17^ we assumed that these could be reduced by half for individuals under self-isolation, consistent with findings that accelerated case isolation in Shenzhen, China, reduced transmission by 35%.^20^ We included regular school closures for holidays in all models, based on national dates for school holidays in England up to Sept 1, 2021, setting school contacts to zero for the duration of these holidays and assuming schools would otherwise stay open unless closure was explicitly modelled.

### Intervention timing and adherence

We set Jan 29, 2020, as the start date of our model (ie, when infections leading to sustained person-to-person transmission begin), which we chose by visually aligning model-predicted deaths to the daily number of COVID-19 deaths reported in the UK^21^ up to March 27 (appendix pp 4, 8). Non-pharmaceutical interventions against previous epidemics—particularly school closures in response to pandemic influenza or severe acute respiratory syndrome (SARS)—have typically been put in place for periods of 1 week to 3 months.^22^ Accordingly, we first evaluated scenarios under which non-pharmaceutical interventions would be deployed for 12 weeks, timed to begin 6 weeks before the peak incidence of new cases for the unmitigated epidemic. When interventions have a short duration, their impact can be influenced by timing. Moreover, if interventions are triggered at the same time across all locations, they can arrive too early in some locations and too late in others. We therefore estimated the impact of triggering interventions at different times—ie by shifting the intervention by 2 weeks, 4 weeks, or 8 weeks relative to the baseline timing—and of triggering each intervention either nationally (ie, with interventions in each county triggered relative to the unmitigated peak incidence across the entire UK) or at a local level (ie, with interventions in each county triggered relative to the unmitigated peak incidence for each specific county).

Adherence to interventions can vary geographically. To estimate the impact of such variation, we simulated the combined intervention (local trigger with a 4-week shift) with varying adherence among counties, with some counties selected at random to show greater adherence and others selected to show less adherence (appendix p 5).

### Further analyses of individual interventions

We modelled the impact of control measures relating to leisure activities in the UK, as an addition to the individual interventions. As other countries in Europe began restricting mass gatherings, there was a question about the impact such measures might have in the UK, with a particular focus on stopping spectator sports.^23^ By analysing the total attendance at spectator sports in the UK, we ran additional simulations to evaluate the potential marginal impact of such restrictions. We also simulated a more general reduction in leisure contacts—which mainly occur in pubs, bars, restaurants, and cinemas—by reducing them by 75%. Previous work on pandemic influenza has estimated that many individuals are likely to choose to avoid such settings, as they perceive them to be risky.^24^

As a sensitivity analysis, we also evaluated the potential impact of schoolchildren being cared for by grandparents on weekdays during school closures, because of concerns over whether this might counteract the benefit of closing schools as a result of higher-risk older adults being exposed to more transmission from children. Specifically, we simulated the introduction of an additional interpersonal contact each weekday between children younger than 15 years and individuals at least 55 years older than the children, for either 20%, 50%, or 100% of all children younger than 15 years.

### Intensive interventions and lockdowns

As well as single 12-week measures, we also analysed the impact of longer-term and repeated interventions. On March 16, 2020, it was announced that a package of intensive interventions would be put in place, including physical distancing, with a particular impact on leisure activities; workers being asked to work from home where possible; shielding of both older individuals (≥70 years) and people in high-risk groups of all ages; school closures; and self-isolation of symptomatic individuals.

Although these intensive interventions would include similar measures to the combined 12-week intervention we had previously modelled, we devised new estimates of their potential impact in light of the specified details of the programme. In particular, we assumed that 30% of workers would be able to work from home,^25^ reducing work and transport contacts among the low-risk general population (assumed to be 90% of adults younger than 70 years) by 30%. We also assumed leisure contacts (which comprise 45% of other contacts^17^) would decrease by 75% in this population. We assumed that work and other contacts would be reduced by 75% among the high-risk general population (which we estimated at 10% of people younger than 70 years) through shielding. Among those aged 70 years or older, we assumed that 75% of work and other contacts would be reduced through shielding; we then further reduced transport contacts (which comprise 11% of other contacts^17^) by 30% to reflect less travel for workers staying at home and less travel for leisure activities, and reduced leisure contacts by 75% (table 1).

Before the announcement of intensive interventions, we had assessed whether shorter, repeated periods of particularly strict restrictions on movement—so-called lockdowns—could be used to supplement a longer-term, more moderate package of interventions, with lockdowns to be deployed as needed to prevent the health system becoming overburdened. Accordingly, we supplemented the modelled intensive interventions with lockdowns phased in when COVID-19 intensive care unit (ICU) bed requirements reached certain national thresholds (1000, 2000, and 5000 beds), which would be kept in place until ICU bed usage fell back below the same trigger threshold, to then be brought in again as needed. We assumed that lockdowns would reduce all contacts outside the home by 90% from their baseline values and would be triggered at a national level rather than at a local level, and that the trigger threshold would not change over time.

### Role of the funding source

Funders had no role in study design, data collection, data analysis, data interpretation, writing of the report, or the decision to submit for publication. The corresponding author had full access to all of the data and the final responsibility to submit for publication.

## Results

**Figure 2:**
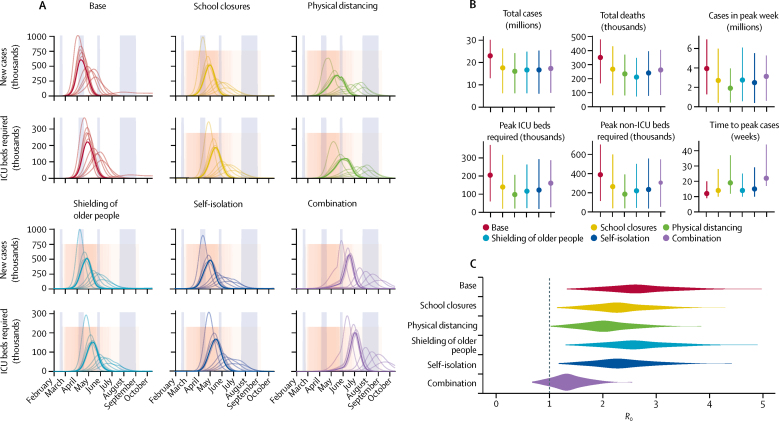
Impact of interventions lasting 12 weeks (A) Daily incidence of new cases and prevalence of ICU beds required over the course of the simulated scenarios in the UK, from February to October, 2020. Divisions on the *x*-axis show the beginning of each calendar month. From 200 realisations of each projection, 11 representative simulations are shown: one for each decile of the total number of cases, with the bold curve showing the simulation resulting in the median projected number of cases. Tall blue shaded regions show scheduled school holiday closures, and pink shaded regions show the distribution of 12-week interventions. (B) Summary of simulated outputs in total number of clinical cases and deaths, clinical cases in the peak week, peak ICU beds required, peak non-ICU beds required, and the time from seeding until the peak of the epidemic. Vertical bars indicate 95% prediction intervals. (C) Estimated distribution of the basic reproduction number, *R*_0_, under each intervention scenario, sampled across all counties and model runs for each scenario. ICU=intensive care unit.

We projected that an unmitigated COVID-19 epidemic would result in a median 23 million (95% PI 13–30) clinical cases in the UK up to December, 2021 (figure 2; appendix p 11). Under this scenario, 85% (95% PI 57–95) of the population would be infected with SARS-CoV-2, with 42% (32–51) of those infected showing clinical symptoms. The unmitigated CFR was 1·5% (1·3–1·7) and the unmitigated infection-fatality ratio was 0·63% (0·45–0·79). In turn, this would result in a projected 350 000 deaths (170 000–480 000) directly attributable to COVID-19, without accounting for any potential increase in the CFR caused by exceeding hospital capacity. The projected peak number of ICU beds required was 200 000 (61 000–370 000). This is roughly 13–80 times ICU capacity in the UK, which we tallied at 4562 beds^26^, ^27^, ^28^, ^29^ in the absence of any efforts to further expand capacity.

When school closures, physical distancing, shielding of older people, self-isolation of symptomatic individuals, and the combination intervention were timed to centre on the peak of the unmitigated epidemic, they each decreased the total number of cases by 20–30% and delayed the peak of the epidemic by 3–8 weeks on average (figure 2A, B). While physical distancing was predicted to have the greatest impact on the total number of cases, shielding of older people was predicted to have the greatest impact on the number of deaths (appendix p 11), because while shielding of older people had a smaller impact on overall transmission, it more effectively protected the highest-risk individuals from infection.

We found that, when implemented alone, none of these shorter-duration interventions were able to decrease health-care need to below available capacity. We estimated that neither school closures, physical distancing, shielding of older people, nor self-isolation alone would reduce *R*_0_ enough to bring about a sustained decline in the incidence of new infections (figure 2C).

Next, we sought to evaluate the potential impact of combining control measures. The most comprehensive of these involves deploying all four individual strategies at the same time. This combination strategy was projected to have a greater impact on *R*_0_ (figure 2C), and in a small proportion (23 [12%] of 200) of simulations was sufficient to halt the epidemic altogether during the intervention period. However, lifting the interventions led to a rapid resurgence of cases in the model, even when *R*_0_ had been kept below 1 during the intervention period (figure 2A).

**Figure 3:**
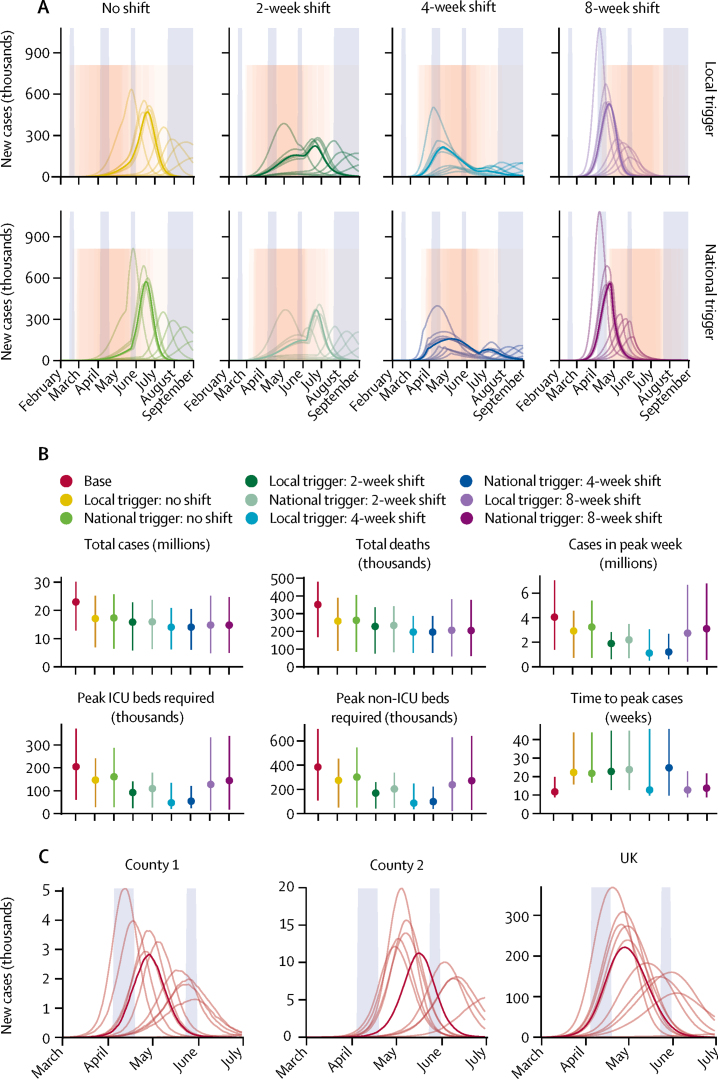
Local versus national triggering and timing of interventions (A) Dynamics of the epidemic under local versus national triggers for introduction of the combined intervention (pink shaded regions). Tall blue shaded regions show regular school holiday closures whereas the pink shaded region shows the intervention period. From 200 realisations of each projection, 11 representative simulations are shown: one for each decile of the total number of new cases, with the bold curve showing the simulation resulting in the median projected daily incidence of cases. (B) Summary of simulated outputs in total number of clinical cases and deaths, clinical cases in the peak week, peak ICU beds required, peak non-ICU beds required, and the time from seeding until the peak of the epidemic. Vertical bars indicate 95% prediction intervals. (C) Illustration of peak timings of new cases varying across two counties in the UK, in comparison with predicted national trends, for a single simulation with no control interventions. Blue shaded regions show regular school holiday closures. Divisions on the *x*-axis in panels A and C show the beginning of each calendar month. ICU=intensive care unit.

We projected that triggering interventions locally instead of nationally could modestly reduce the total number of cases and deaths, as well as reduce peak demands on the health-care system (figure 3A, B; appendix p 12). However, our simulations do not account for any differences in the implementation of or adherence to control measures that might arise from any lack of coordination at a national level should the timing of interventions vary in different parts of the country. Examining the simulated dynamics at a county level shows that the timing of local epidemics can vary among counties, and highlights that epidemics at a local level are predicted to peak more sharply than they do across the entire UK (figure 3C).

When only a single, 12-week intervention is deployed, our projections also showed that instead of centring measures over the unmitigated peak of cases, it was preferable to trigger the intervention later to reduce the total health burden (figure 3B). This is because the introduction of control measures itself shifts the peak later in time—ie, by flattening the curve of the epidemic—and therefore the optimal timing of the intervention is also delayed (figure 3A). In particular, the most effective timing for introduction of measures could involve a delay of as much as 4 weeks (figure 3B). However, optimally timing an intervention could be more difficult in practice than these scenarios suggest, since here they are run with complete knowledge of when the simulated peak would occur in the absence of any intervention.

When varying adherence to interventions among counties, median outcomes were similar but less certain: where projections showed 14 million (95% PI 6·2–21) cases and 200 000 deaths (80 000–290 000) by December, 2021, without between-county variation, they showed 14 million (6·0–21) cases and 200 000 deaths (77 000–300 000) with county-level variation (appendix p 14).

We estimated that, in comparison with other potential interventions, a ban on spectator sports from March 17 to Sept 1, 2020, would have a relatively small impact on the total number of cases, namely resulting in 15 000 fewer cases (95% PI 110 000 fewer cases to 70 000 more cases) up to Sept 1 (appendix p 6). Although yearly attendance at sporting events is high (75·1 million spectators per year^30^), even if we assume that people make the equivalent of their mean daily physical contacts at such events (ie, five contacts per person, to make a total of 375 million), this number is very low relative to the number of yearly contacts that occur outside the context of sporting events (269 billion^17^). A more general reduction in leisure contacts by 75% was estimated to have a more substantial impact on the epidemic, reducing cases by 1·9 million (0·37–4·6) up to Sept 1. In other words, while banning spectator sports might have decreased the total number of cases, we estimated that other potential policies relating to leisure activities would probably be more effective.

When considering the potential impact of children being cared for by grandparents, we found that, over a period of school closure from March 17 (ie, after the intensive interventions package was announced) to July 20, 2020, one additional contact per weekday between children younger than 15 years and an older individual could, in the worst case—ie, at the upper limit of the 95% PI—almost entirely eliminate the benefit of closing schools in terms of the number of deaths and peak ICU bed occupancy. Specifically, closing schools reduced deaths from 120 000 (95% PI 380–270 000) to 65 000 (150–260 000) and reduced peak ICU beds required from 53 000 (190–170 000) to 29 000 (580–140 000) up to July 20, but introducing one additional contact per weekday between children and older individuals for all children increased deaths to 79 000 (160–290 000) and increased peak ICU beds required to 35 000 (560–160 000) up to July 20 (appendix p 13).

**Figure 4:**
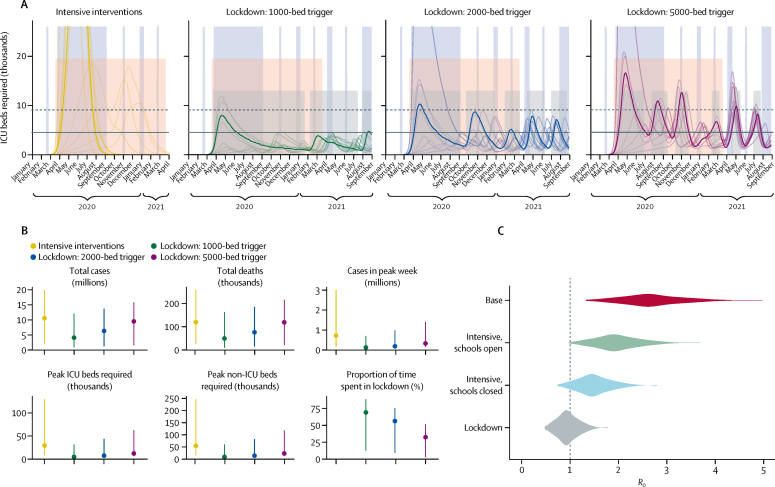
Projected impact of intensive control measures with reactive lockdowns (A) Dynamics of the epidemic under different triggers for introduction and lifting of lockdowns (median timing of lockdowns shown as low grey shaded areas). Divisions on the *x*-axis show the beginning of each calendar month. From 200 realisations of each projection, 11 representative simulations are shown: one for each decile of the total number of ICU beds required, with the bold curve showing the simulation resulting in the median projected ICU bed requirement. Horizontal guides show the estimated number of ICU beds in the UK as of January, 2020 (solid line), and with a hypothetical doubling of capacity (dashed line). Tall blue shaded regions show school closures whereas the pink shaded region shows a background period of intensive interventions. Dynamics are shown up to April, 2021 (intensive interventions) or September, 2021 (lockdown scenarios), but all scenarios were modelled up to the end of December, 2021. (B) Summary of simulated outputs in total number of clinical cases and deaths, clinical cases in the peak week, peak ICU beds required, peak non-ICU beds required, and the time from seeding until the peak of the epidemic. Vertical bars indicate 95% prediction intervals. (C) Estimated distribution of the basic reproduction number, *R*_0_, under three different interventions—intensive physical distancing with schools open and closed, and lockdown—sampled across all counties and model runs for each scenario. ICU=intensive care unit.

When modelling the likely impact of the proposed intensive interventions strategy, we found implementation of these measures had the potential to delay the peak of the epidemic by 7 weeks (95% PI 1–49), from 12 weeks (9–20) to 19 weeks (10–69) after the start of community transmission, and to reduce the total number of deaths by half (figure 4A; table 2). Despite this substantial reduction in burden, the projections still showed a large number of cases (1·9–20 million), and a large number of ICU beds (8300–130 000) occupied during the peak of the epidemic (figure 4B; table 2). Indeed, we projected that ICU bed capacity could be exceeded by five times or more for several weeks. While we did not explicitly predict the impact of this on mortality rates, this would almost certainly lead to a substantially increased CFR.

**Table 2:** Projected impact of intensive control measures and lockdown in the UK

	**Intensive interventions**	**Lockdown with 1000-bed trigger**	**Lockdown with 2000-bed trigger**	**Lockdown with 5000-bed trigger**
Total cases, millions*	11 (1·9–20)	4·1 (0·85–12)	6·3 (1·2–14)	9·5 (1·5–16)
Total deaths*	120 000 (27 000–260 000)	50 000 (9300–160 000)	76 000 (15 000–190 000)	120 000 (22 000–220 000)
Cases in peak week	720 000 (170 000–3 000 000)	120 000 (46 000–700 000)	180 000 (86 000–980 000)	330 000 (160 000–1 400 000)
Deaths in peak week	8300 (2300–37 000)	1400 (510–9000)	2100 (930–13 000)	3400 (1800–17 000)
Peak ICU beds required	29 000 (8300–130 000)	4900 (1800–32 000)	7500 (3500–44 000)	12 000 (6700–62 000)
Peak non-ICU beds required	55 000 (15 000–250 000)	9100 (3600–60 000)	14 000 (6800–83 000)	23 000 (13 000–120 000)
Time to peak cases, weeks	19 (10–69)	60 (8–92)	60 (8–72)	35 (8–69)
Time spent in lockdown (Jan 29, 2020—Dec 31, 2021)	..	69% (13–88)	56% (9·2–76)	33% (2·8–52)
Total infected, millions*	27 (5·3–46)	11 (2·1–29)	17 (3·0–33)	25 (4·4–38)

Data are median (95% prediction interval) and are given to two significant figures. Time to peak cases is measured from Jan 29, 2020. Totals are calculated up to Dec 31, 2021. ICU=intensive care unit.

* Simulations were run to Dec 31, 2021, so reported total cases, deaths, and infections under the lockdown projections do not capture any cases, deaths, or infections occurring after this point.

We found that adding periods of lockdown to the intensive interventions scenario, to be triggered when ICU beds required for patients with COVID-19 exceeded a threshold of either 1000, 2000, or 5000 beds nationally, would still result in a high number of ICU beds being occupied, but at much lower levels than the scenario without lockdowns (figure 4A). Lockdown periods were sufficient to bring *R*_0_ near or below 1 (figure 4C), and hence to bring about a decrease in the incidence of new infections. We found that, depending on the threshold ICU bed occupancy at which lockdown periods were triggered, there was a tradeoff between having fewer, longer lockdown periods (lower threshold) and having more frequent, shorter lockdown periods (higher threshold), with the higher thresholds resulting in less time spent in lockdown overall but higher peak demands on ICU bed capacity (figure 4; table 2). Lower thresholds also resulted in more individuals remaining susceptible at the end of the simulation period (table 2), potentially increasing the total duration for which recurrent lockdowns would need to be maintained. The recurrent lockdown scenario with the most stringent triggering threshold of 1000 ICU beds occupied by COVID-19 patients reduced total COVID-19 deaths by 58% (95% PI 30–80) relative to the intensive interventions scenario, and by 86% (64–96) relative to the unmitigated epidemic.

## Discussion

Using an age-structured transmission dynamic model, we explored different scenarios for COVID-19 transmission and control in the general population of the UK. We found that moderate interventions lasting for 12 weeks, such as school closures, self-isolation of symptomatic individuals, or shielding of older people, would probably not have been sufficient to control the epidemic and to avoid far exceeding available ICU capacity, even when these measures were used in combination. In particular, school closures had little effect in our projections, despite our model accounting for substantial asymptomatic transmission among children.^16^ This contrasts with strategies aimed at suppressing the spread of pandemic influenza, for which school closures are often a key intervention.^16^, ^31^ However, we estimated that a scenario in which more intense lockdown measures were implemented for shorter periods, against a general background of physical distancing measures, might be able to keep projected case numbers at a level that would not overwhelm the health system. These findings are consistent with studies that explored subsets of these control measures for COVID-19 in the UK,^11^ France,^15^ the USA,^12^ and Canada.^13^ However, we integrate model trajectories over a distribution of values for *R*_0_ and seeding dates to provide uncertainty bounds for our projections, explore the impact of alternative timings of interventions, and account for variation in the proportion of symptomatic cases by age as estimated from case data.^16^ Directly comparing these projections to the ongoing COVID-19 epidemic in the UK is complicated because enacted control measures have not exactly followed the scenarios outlined here. However, as a point of comparison, recent empirical estimates of the reproduction number in the UK^32^, ^33^ are compatible with our assumptions concerning *R*_0_ and the impact of lockdown measures (appendix p 13).

The model presented here is subject to several limitations. Because the model does not explicitly structure individuals by household, we are unable to evaluate the impact of measures based on household contacts, such as household quarantine, where all members of a household with a suspected COVID-19 case remain in isolation. Such contact-targeted measures could increase the impact of a package of interventions by limiting spread in the community. However, the presence of asymptomatic infections^34^ means that isolation based on symptomatic case identification would be unlikely to fully prevent ongoing transmission. We also do not explicitly include individual-level variation in transmission (ie, so-called superspreading events^35^), although if considered at the individual level, the processes underlying the model would generate substantial variation from case to case. There are several examples of such events for COVID-19,^36^ and individual-level variation is probably important in influencing the success of control measures in the very early stages of an outbreak.^5^ However, as outbreaks of directly transmitted infections become larger, the population-level dynamics will predominantly be driven by the average mixing pattern between key epidemiological groups, particularly between different ages.^16^, ^37^ We therefore used a stochastic model to capture variation in these population-level dynamics. Our proections focus on COVID-19 transmission in the general population of the UK, and so do not account for health-care-associated transmission and the interventions appropriate for controlling transmission in health-care settings. We also assumed that individuals would be immune after infection for at least 1–2 years (ie, the duration of the period considered). Duration of antibody responses to SARS coronavirus lasts for 2–3 years in most patients,^38^ and modelling has suggested that SARS-CoV-2 could enter into regular circulation if immunity is not permanent.^12^ However, the latter study suggests that short-term projections (ie, approximately 1 year), such as those presented here, are relatively insensitive to assumptions about the duration of immunity.

We also assumed that subclinically infected individuals were 50% as infectious as clinical cases. A study of 2147 close contacts in Ningbo, China, estimated that the mean onward transmission from asymptomatic infections was 65% (95% high density interval 20–120) that of symptomatic cases.^34^ However, symptomatic cases were found to be more likely to generate new symptomatic infections compared with asymptomatic infections. This suggests that the overall relative contribution of asymptomatic individuals to new infections might be lower than 65%, and hence 50% is a plausible assumption. We used mixing matrices for the UK measured in 2006,^17^ and changes in contact patterns since then might alter the potential effect of interventions. The length of stay in ICU and the fractions of hospitalisation, ICU use, and death are estimated using data from China, and differences in UK populations could affect our estimates of health-care demand.

Finally, projections for the relative impact of the various physical distancing measures explored here are estimates only. The COVID-19 epidemic is without precedent in recent history, so it was not possible to make substantially data-driven assumptions concerning the impact of non-pharmaceutical interventions on contact patterns. It remains difficult to estimate the relative impact of potential constituent interventions even now, since a substantial package of interventions was implemented in short succession across the UK. Nonetheless, there is some indication that our assumptions concerning the impact of full-population lockdown are consistent with measurements of the effective *R*_0_^5^ and of contact rates between individuals^33^ in the lockdown period (appendix p 13). We are also unable to explicitly decompose physical distancing measures into constituent components, such as staying 2 m apart, increased hand washing, and face mask wearing, as the relative effectiveness of these components has not yet been estimated. While our analysis shows that periodic lockdowns could substantially reduce the burden of COVID-19 without measures being in place indefinitely, there are likely to be better strategies for selecting the timing and duration of lockdowns than those explored here.

The results we present here summarise the key analyses and scenarios we presented to decision makers over February–March, 2020, which evolved continuously as new information became available. A reasonable worst-case scenario with and without school closures, focusing on Birmingham as an illustrative example, was presented to the Scientific Pandemic Influenza Group on Modelling, which gives expert advice to the UK Department of Health and Social Care and wider UK Government, on Feb 26, 2020. This was followed by an exploration of national-level impact of shorter-duration interventions (as in figure 2) presented on March 2, 2020, which explored various assumptions concerning intervention length and efficacy. We expanded our analysis to explicitly cover all counties in England and analysed the timing of measures, and local versus national deployment of interventions (as in figure 3), on March 8, 2020. Our analyses of the impact of curtailing sporting events and leisure activities (as in appendix p 6), and of the potential impact of repeated lockdown measures (as in figure 4), were presented on March 11, 2020. Our sensitivity analysis for increased child–grandparent contacts (as in appendix p 6) was presented on March 17, 2020. The results shown in this Article are based on an updated version of the model and reflect our current state of knowledge about the transmission dynamics of COVID-19. However, our overall conclusions about the relative effectiveness of different strategies for reducing the burden of COVID-19 in the UK are the same as those presented to decision makers in real time.

## Data sharing

All analysis code and data are available on the CMMID COVID-19 GitHub page.
