# Supplementary material for: Effects of non-pharmaceutical interventions on COVID-19 cases, deaths, and demand for hospital services in the UK: a modelling study
Source: Lancet Public Health. 2020 Jun 2;5(7):e375–85. doi: 10.1016/S2468-2667(20)30133-X (PMC7266572; doi:10.1016/S2468-2667(20)30133-X)
Supplement: Supplementary appendix [file mmc1.pdf]

THE LANCET
Public Health

Supplementary appendix

This appendix formed part of the original submission. We post it as supplied by the authors.

Supplement to: Davies NG, Kucharski AJ, Eggo RM, et al, on behalf of the Centre for the Mathematical Modelling of Infectious Diseases COVID-19 working group. Effects of non-pharmaceutical interventions on COVID-19 cases, deaths, and demand for hospital services in the UK: a modelling study. *Lancet Public Health* 2020; published online June 2. hhttps://doi.org/10.1016/S2468-2667(20)30133-X.

Supplementary information

Dynamic transmission model 2

Algorithmic details of transmission model 2

Key parameters of the transmission model 3

Hospital burden estimation 4

Derivation of contact rates for the “Intensive Interventions” scenario 5

Scenario with varying adherence from county to county 5

Analysis of ban on spectator sports 5

Supplementary figures 6

- Figure S1. Impact of reducing leisure events and impact of increased childcare from older age groups 6
- Figure S2. R_0 distribution used 7
- Figure S3. Projected deaths versus reported deaths 8
- Figure S4. Burdens under the “combined” intervention with national triggering and a 4 week shift (see main text, Figure 3), without and with county-level variability in adherence 9

Supplementary tables 10

- Table S1. Model parameters. 10
- Table S2. Age-specific hospitalisation and fatality risk. 11
- Table S3. Projected impact of 12-week interventions in the UK 11
- Table S4. Projected impact of control measures in the UK depending upon local versus national triggering and according to shift from centring on predicted peak. 12
- Table S5. Projected impact of control measures relating to leisure activities in the UK. 13
- Table S6. Projected impact of school closures, depending upon additional contact between children and older age groups, in the UK. 13
- Table S7. Comparison of projected versus empirically-estimated reproduction numbers in the UK. 13
- Table S8. Projected impact of control measures in the UK depending upon whether adherence is the same across all counties or varies from county to county. 14

Supplementary references 15

Centre for Mathematical Modelling of Infectious Diseases (CMMID) COVID-19 working group 17

Dynamic transmission model

We analyse a stochastic compartmental model (**Fig. 1**) stratified into 5-year age bands, with time approximated in discrete 6-hour steps. The model tracks 66.4 million UK residents aggregated to the 186 county-level administrative units across England, Wales, Scotland, and Northern Ireland. We run 200 stochastic realizations for each projection.

We assume that the population initially consists of susceptible individuals (S), who become exposed (E) after effective contact with an infectious person. After an incubation period lasting 4 days on average, exposed individuals of age i will develop either a clinical infection with probability y_i, or a subclinical infection with probability $1 - y_i$. Clinical cases begin with a preclinical but infectious (I_P) state lasting 1.5 days on average; these individuals then progress to a clinically infected state (I_C), which we assume marks the onset of a clinical case. We assume that subclinical infections (I_S) are half as transmissible as preclinical and clinical infections. Regardless of whether they are clinically or subclinically infected, individuals remain infectious for 5 days on average and are then removed (R) from the infectious state; we assume that removed individuals are immune to reinfection over the 1–2 years over which we simulated the epidemic. Hospitalisations and deaths from COVID-19 are assumed to occur among clinical cases only, and we assume that the clinical outcome of a case does not impact upon transmission dynamics.

The amount of time a given individual spends in states E, I_P, I_C, or I_S is drawn from distributions d_E, d_P, d_C, or d_S, respectively (**Table S1**). We assume that all durations are gamma distributed for simplicity. The force of infection for an individual in age group i at time t, where t is defined in 6 hour time steps,

$$\lambda_{i,t} = u \sum_j c_{ij,t} (I_{Pj} + I_{Cj} + f I_{Sj}) / N_j, \quad (1)$$

is the rate at which susceptible individuals enter the exposed state. Here, u is an individual's susceptibility to infection upon contact with an infectious person, $c_{ij,t}$ is the number of age-j individuals contacted by an age-i individual per day at time t, f is the relative infectiousness of a subclinical infection, and $(I_{Pj} + I_{Cj} + f I_{Sj}) / N_j$ is the effective probability that a random age-j individual is infectious.

To calculate the basic reproductive number, R_0, defined as the average number of secondary infections generated by a typical infectious individual in a fully susceptible population, we define the ith-row and jth-column element of the next generation matrix as

$$NGM_{ij} = u c_{ij,t} (y_j E(d_P + d_C) + (1 - y_j) f E(d_S)) ,$$

where E denotes the expectation. Then, R_0 is the absolute value of the dominant eigenvalue of the next generation matrix.

Algorithmic details of transmission model

Focusing on a single county, the number of age-i individuals in the susceptible and recovered compartments are notated as S_i and R_i, respectively, and the number of age-i individuals with d time steps remaining in the exposed, preclinical, clinical, and subclinical compartments are notated as $E_{i,d}$, $I_{Pi,d}$, $I_{Ci,d}$, and $I_{Si,d}$, respectively, where the index d runs from 0 to D = 240 (i.e. the maximum duration in any of these compartments is 60 days). At simulation start, S_i is equal to the total number of age-i individuals in the county, and all other compartments are set to 0. The model proceeds in time steps of size Δt = 0.25 days. The underlying model uses integer arithmetic, which reduces the risk of numerical instability owing to inaccuracies in floating-point calculations. Below, when an individual is added to compartments E_i, I_{Pi}, I_{Ci}, or I_{Si}, this means that a duration d is first drawn from the discrete distribution d_E, d_P, d_C, or d_S, respectively (see Table S1), and then the number of individuals with d time steps remaining in the corresponding compartment ($E_{i,d}$, $I_{Pi,d}$, $I_{Ci,d}$, or $I_{Si,d}$) is incremented by one, for each individual being added to the compartment. In other words, the length of time any given individual will spend in compartments E_i, I_{Pi}, I_{Ci}, or I_{Si} is determined randomly at the point that the individual enters that compartment.

At the beginning of each time step, any changes to simulation parameters resulting from the introduction or release of interventions are made. Then, if the number of new individuals to be seeded externally into the county is X, then X random age groups are chosen uniformly with replacement and one individual is added to the compartment E_i, and removed from the compartment S_i, for each randomly-chosen age group for seeding. Then, to represent new infections via transmission, $Y_i \sim binomial(n = S_i, p = 1 - exp\,(-\lambda_i \Delta t))$ individuals are added to compartment E_i and removed from compartment S_i, where λ_i is the force of infection as defined in equation (1) above, for each age group i. For compartments E_i, I_{Pi}, I_{Ci}, and I_{Si}, individuals with 0 time steps remaining in the compartment are "matured" into the next compartment (following the transitions shown in Fig. 1 of the main text). For example, for the transition $I_P \rightarrow I_C$, for each age group i, $I_{Pi,0}$ individuals are added to compartment I_{Ci}, then we set $I_{Pi,z} \leftarrow I_{Pi,z+1}$ for each $z \in \{0,1,2,\ldots,D-1\}$ and set $I_{Pi,D} \leftarrow 0$ to move each individual remaining in state I_P one step closer to moving to state I_C. For the transition from the exposed state to either the preclinical or subclinical state, $W_i \sim binomial(n = E_{i,0}, p = y_i)$ individuals are added to the preclinical compartment I_{Pi} and $E_{i,0} - W_i$ individuals are added to the subclinical compartment I_{Si} for each age group i.

To track hospital bed occupancy and deaths, we also track additional states H_I (holding state for individuals who will enter ICU), H_G (holding state for individuals who will enter the general ward), B_I (currently occupied beds in ICU), B_G (currently occupied beds in general ward) and D (holding state for individuals who will die). These states do not directly impact upon transmission dynamics, and are populated as follows. For each age group i, if V_i age-i individuals are entering the clinical compartment I_{Ci}, then this represents case onset and the random vector variate $(S_i, T_i, U_i) \sim multinomial(n = V_i, p = (P_{Ii}, P_{Gi}, 1 - P_{Ii} - P_{Gi}))$ is drawn (where P_{Ii} is the probability that a symptomatic age-i individual enters the ICU and P_{Gi} is the probability that a symptomatic age-i individual enters the general ward); then, S_i individuals are added to compartment H_{Ii}, and T_i individuals are added to the compartment H_{Gi}. Durations spent in these "holding" compartments are drawn from the delay from onset to hospitalisation distribution in Table S1. Individuals eventually "mature" from state $H_I \rightarrow B_I$ and from state $H_G \rightarrow B_G$, such that compartments B_I and B_G track the number of individuals currently in an ICU bed or a general ward bed; durations spent in these compartments are drawn from the distributions of duration of hospitalisation in ICU and non-ICU beds in Table S1. To track deaths, $Q_i \sim binomial(n = V_i, p = P_{Di})$ individuals are added to the compartment D_i, where P_{Di} is the case fatality ratio for age-i individuals; time to death (i.e., exit from the D_i compartment) is drawn from the distribution of delay to death from Table S1, and deaths are recorded when individuals mature out of the D_i state.

These steps are repeated for each county and for each time step from simulation start to simulation end.

For regular school closures, we assume that schools are closed, with school contacts set to zero, for the periods of: 16/02/2020 - 22/02/2020; 05/04/2020 - 18/04/2020; 24/05/2020 - 30/05/2020; 22/07/2020 - 01/09/2020; 25/10/2020 - 31/10/2020; 20/12/2020 - 02/01/2021; 14/02/2021 - 20/02/2021; 01/04/2021 - 17/04/2021; 30/05/2021 - 05/06/2021; and 25/07/2021 - 01/09/2021. These are based upon periods of school holidays for England (source: https://www.gov.uk/school-term-holiday-dates).

Key parameters of the transmission model

Published studies suggest a serial interval of approximately 6.5 days[1,39,40], and suggest that the length of the preclinical period is approximately 30% of the total period of clinical infectiousness [19]. From this, we fixed the mean of the latent period to 4 days, the mean duration of preclinical infectiousness to 1.5 days, and the mean duration of clinical infectiousness to 3.5 days.

We sampled the basic reproduction number R_0 from a consensus distribution (**Fig. S1**) derived from published sources available at the time projections were made. For each reported value of the basic reproduction number, we matched a flexible PERT distribution (a shifted beta distribution parameterised by minimum, maximum, and

mode) to the median and confidence interval reported in each study. We sampled across all studies, with each study weighted equally. The distribution overall has a mean R_0 of 2.68, with a standard deviation of 0.57. To capture this mean and variance with a simple parsimonious function, we used a normal distribution with these parameters for our simulations. In Figs. 2c, and 4c of the main text, plotted R_0 distributions represent the population-weighted R_0s in each county across all 200 simulations.

To capture observed differences by age in developing COVID-19 symptoms upon infection, the age-specific clinical fraction y_i was adopted from our estimate based on cases from 6 countries[16]. The relative infectiousness of subclinical cases, f, was assumed to be 50% relative to clinical cases, as we assumed in a previous study[16].

We used the Office for National Statistics data on the population by age for each of 186 county-level subdivisions of the UK[41], comprising non-metropolitan counties, metropolitan counties, unitary authorities and London boroughs in England; unitary authorities in Wales; council areas in Scotland; and local government districts in Northern Ireland (hereafter referred to as "counties"). We used contact data from the POLYMOD study[17] and the R package socialmixr[42] to generate age-stratified contact matrices for the UK, generating separate contact matrices for each county according to the population structure for that county, assuming that the number of age-j contacts made by an age-i individual scales with the number of age-j individuals in a county.

We assumed that epidemics in each county are seeded by 2 individuals per day for 28 days; after this point, further seeding has very little impact owing to extensive community transmission. Increasing (decreasing) the number of seed individuals has a similar impact on later model dynamics as making the epidemic start time earlier (later). Seeding times are staggered so that London boroughs are seeded on a random day in the first week of the simulation, and other locations are seeded on a random day in the first four weeks of the simulation. We assume that transmission between counties is negligible, instead allowing the staggered seeding of infection among counties to simulate the process of gradual introduction of the epidemic across the UK. The start date of the model is 29th January 2020, which we chose by visually aligning model-predicted deaths to the daily number of COVID-19 deaths reported in the UK[43] up to 27th March (Fig. S2).

Each realisation of the model within a set of 200 simulations is given a random set of seeding times and a random value for R_0 as detailed above, with the same set of 200 values for R_0 and per-county seeding times maintained across different scenarios to ensure scenarios are comparable.

Hospital burden estimation

To calculate ICU and non-ICU beds in use through time, we scaled age-stratified symptomatic cases by age-specific hospitalisation and critical outcome probability, then summed to get the total number of hospitalised and critical cases. We then distributed hospitalised cases over time based on expected time of hospitalisation and duration admitted. We assumed gamma-distributed delays, with the shape parameter set equal to the mean, for: delay from symptom onset to hospitalisation of mean 7 days (standard deviation 2.65)[44,45]; delay from hospitalisation to discharge / death for non-ICU patients of mean 8 days (s.d. 2.83)[46]; delay from hospitalisation to discharge / death for ICU patients of mean 10 days (s.d. 3.16)[45]; and delay from onset to death of mean 22 days (s.d. 4.69)[44,45].

We calculated the age-specific case fatality ratio (Table S2) based on data from the COVID-19 outbreak in China[4]. We first calculated the naïve case fatality ratio, nCFR, (i.e. deaths/cases) for each 10-year age group, then scaled down the naïve CFR to adjust for case underascertainment, multiplying the naïve age-stratified CFR estimates by 0.52—which was previously estimated from data from the Diamond Princess cruise ship[19]—to give an adjusted CFR. We then calculated risk of hospitalisation based on the ratio of severe and critical cases to cases (18.5%) and deaths to cases (2.3%) in the early China data, which we took to imply 8.04 times more hospitalisations than deaths in each age group. We assumed all age groups had a 30% risk of requiring critical care if hospitalised[45]. The age-specific case fatality ratio and hospitalisation rates were calculated in 10-year age bands, which we applied to both 5-year age groups within a given band.

Derivation of contact rates for the "Intensive Interventions" scenario

For the "Intensive Interventions" scenario, we assumed that 30% of workers would be able to work from home[25], reducing work and transport contacts (11% of "other" contacts) among the low-risk general population (assumed to be 90% of adults under 70) by 30%. We also assumed leisure contacts (45% of "other" contacts) would decrease by 75% in this population. We assumed that work and "other" contacts would be reduced by 75% among the high-risk general population (10% of under-70s) through shielding. Among those aged 70 and above, we assumed that 75% of work and other contacts would be reduced through shielding; we then further reduced transport contacts by 30% and leisure contacts by 75%.

Scenario with varying adherence from county to county

Our projections in the main text assumed that the reduction in contact rates associated with interventions would be the same in all counties. In reality, there could be regional differences in the relative impact of physical distancing interventions, which may have an impact on model dynamics. To evaluate this possibility, we ran a sensitivity analysis for the "combined" intervention triggered locally with a 28-day shift (**Fig. 3**).

We assumed the reduction in contacts and infectiousness of symptomatic individuals was drawn from a beta distribution centered on the overall reduction when counties did not vary. Specifically, for each county we first drew a random number $A \sim Uniform(0,1)$ representing the "adherence index" of the county. In place of 0.5 for work and "other" contacts in under-70s, we used $qbeta(A, \alpha = 20 \cdot 0.5, \beta = 20 \cdot (1 - 0.5))$, where qbeta is the beta distribution inverse CDF. Similarly, in place of 0.25 for work and "other" contacts in over-70s, we used $qbeta(A, \alpha = 20 \cdot 0.25, \beta = 20 \cdot (1 - 0.25))$, and for the infectiousness of symptomatic individuals due to self-isolation we used $qbeta(A, \alpha = 20 \cdot 0.65, \beta = 20 \cdot (1 - 0.65))$. We used the same value of A for each of these three measures to reflect an assumption that if adherence was lower in a given county, it would be lower for all three effects of the intervention. Results were broadly similar (**Fig. S3, Table S8**), although 95% prediction intervals were wider, peak burdens were slightly lower, and total burdens were marginally higher when adherence varied among counties. Subsequent analysis of UK mobility data[47] suggests that the actual variation in adherence between counties is relatively minor (**Fig. S3d**).

Analysis of ban on spectator sports

Our analysis of a potential ban on spectator sports between 17 March and 1 September 2020 found a relatively small impact upon transmission, with a median impact of 15 000 fewer cases (95% prediction interval: 110 000 fewer to 70 000 more cases) over this period. The prediction that banning spectator sports could result in an increase in the total number of cases results from an interaction between the ban on spectator sports and periods of regular school closure. Small shifts in epidemic dynamics, such as those caused by the ban on spectator sports, can result in other, larger changes in contact patterns—here, those resulting from regular school closures—having a relatively larger or smaller impact upon overall epidemic dynamics. We did not model a potential increase in the number of contacts in other settings (such as pubs) that might result from attendance at stadium sports being banned in isolation; conversely, we also did not model the potential impact of banning spectator sports on public awareness of the epidemic. The aim of this analysis was not to argue that spectator sports should not be banned, or to argue that a potential reduction in total cases by 15 000 was insignificant. Rather, the aim was to show that this measure, taken in isolation, was unlikely to have a substantial impact upon COVID-19 cases, relative to other potential policy decisions.

Supplementary figures

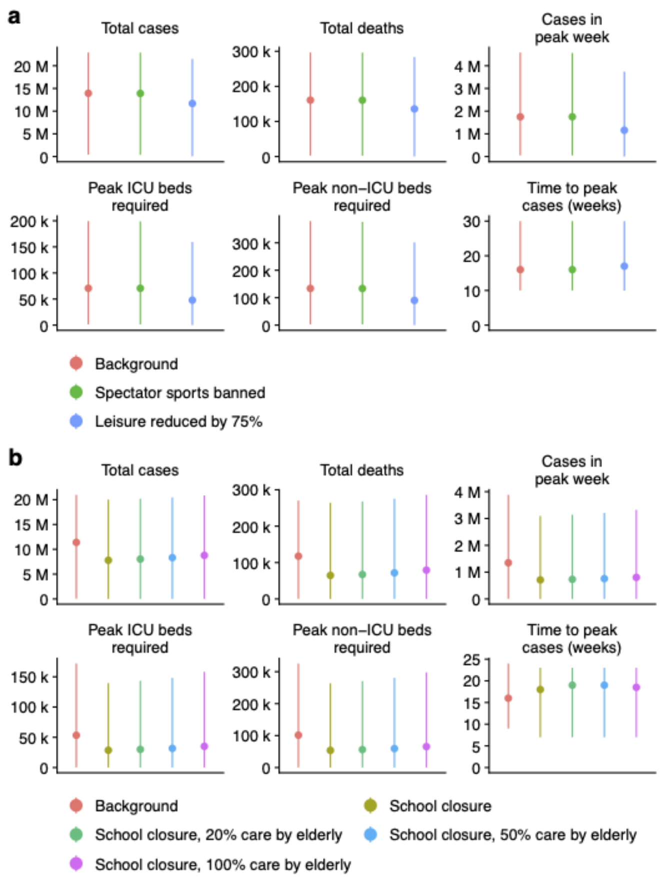

Figure S1. Impact of reducing leisure events and impact of increased childcare from older age groups

(a) Effect of banning spectator sports, and decreasing leisure activities on the total cases, total deaths, and peak number of cases, ICU beds, non-ICU beds, and the time to peak week in the simulated epidemics. The "Background" to which these interventions are compared is school closures plus shielding of older age groups. (b) Effect of varying increases in contacts between children and older adults during school closures and effect on the total cases, total deaths, and peak number of cases, ICU beds, non-ICU beds, and the time to peak week in the simulated epidemics. The "Background" to which these interventions are compared is the "Intensive Interventions" of Table 12, without any additional period of school closure.

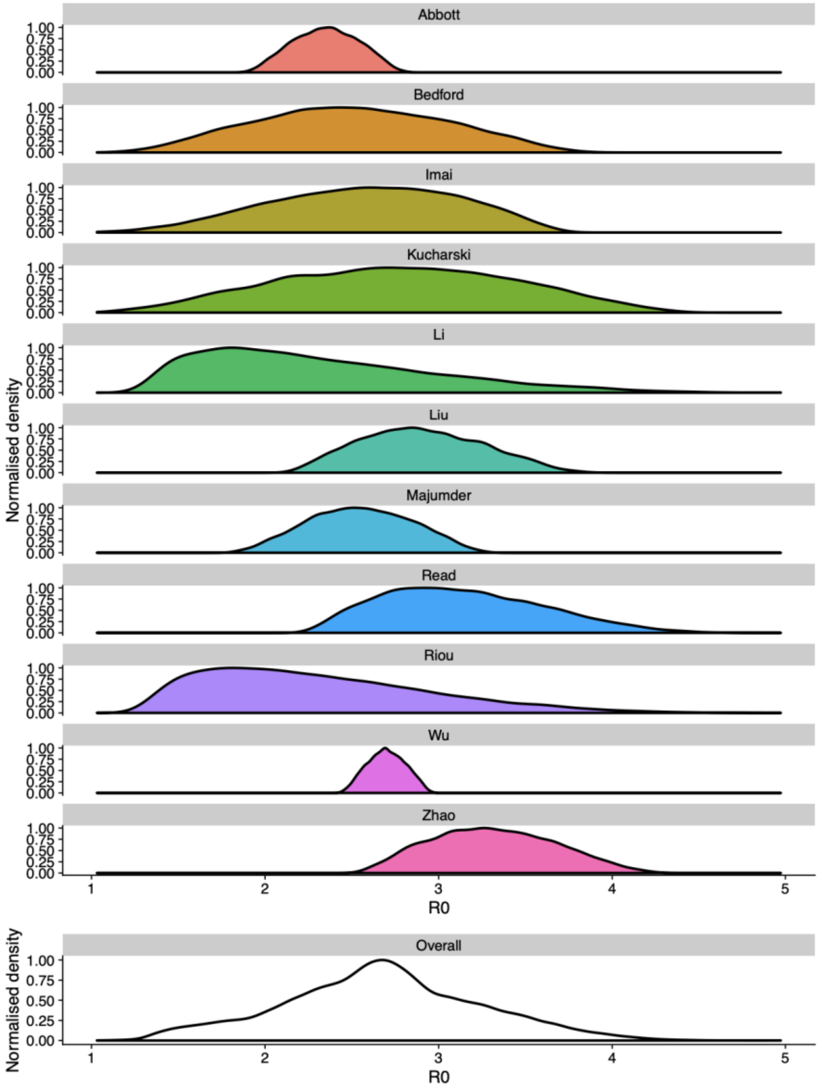

Figure S2. $\boldsymbol{R_0}$ **distribution used**

Sources used can be found among refs. 1, 2, 6, 48–55.

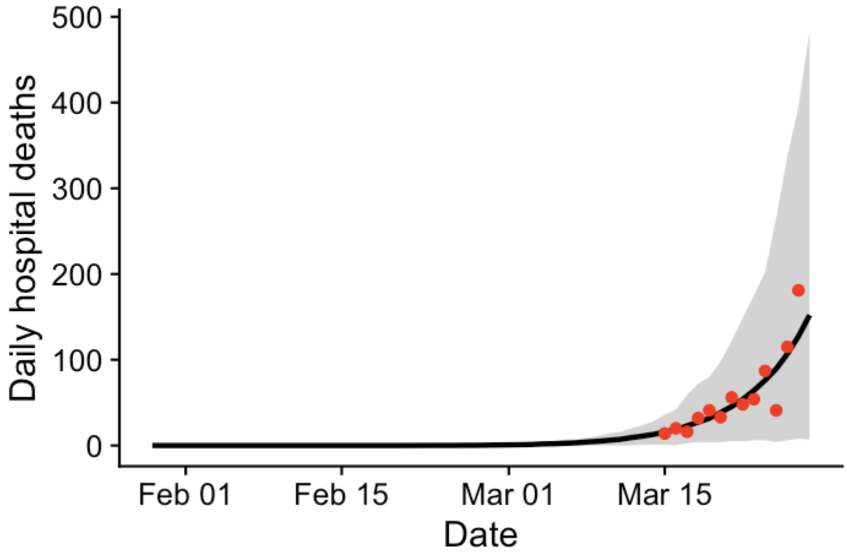

Figure S3. Projected deaths versus reported deaths

Deaths projected under "**Base**" scenario (line and grey ribbon: mean and 95% prediction interval) plotted against reported deaths in the UK (red points) up to March 27th; visual alignment was used to select an earliest starting date of January 29th, 2020 for epidemic seeding.

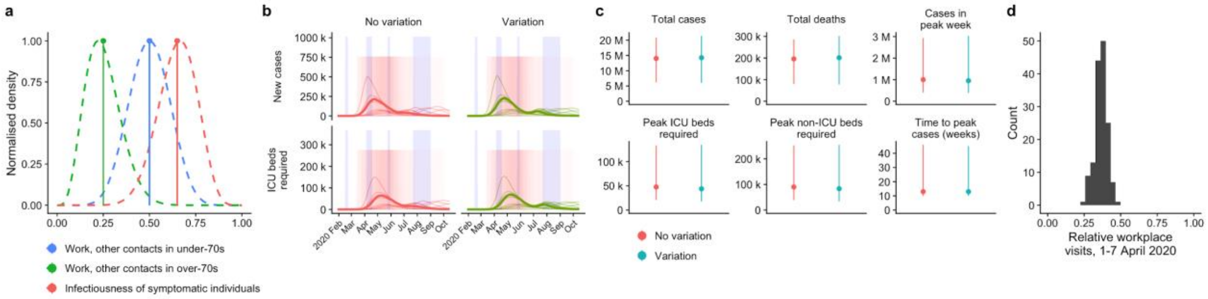

Figure S4. Burdens under the "combined" intervention with national triggering and a 4 week shift (see main text, Figure 3), without and with county-level variability in adherence

(a) Work and "other" contacts for under- and over-70s, and the infectiousness of symptomatic individuals, was fixed at a single value in the main text analysis (solid lines). Here, we evaluate the impact of drawing these values from a beta distribution for each county, with the same mean for each parameter but with substantial variability around the mean. **(b, c)** Overall, results are similar regardless of whether adherence in each county varies or not, although when there is variability among counties, 95% prediction intervals are slightly wider, overall cases and deaths are slightly higher, and peak cases and deaths are slightly lower. See also **Table S7**. **(d)** Subsequent analysis of mobility data for the UK[47] suggests that the relative frequency of workplace visits in the first 7 days of April shows small differences at the county level, suggesting that the actual between-county variation in adherence during "lockdown" measures has been relatively minor.

Supplementary tables

Table S1. Model parameters.

Parameter	Description	Value	Reference
d_E	Latent period (E to I_P and E to I_S; days)	$\sim gamma(\mu = 4.0, k = 4)$	1, 39, 40
d_P	Duration of preclinical infectiousness (I_P to I_C; days)	$\sim gamma(\mu = 1.5, k = 4)$	19
d_C	Duration of clinical infectiousness (I_C to R; days)	$\sim gamma(\mu = 3.5, k = 4)$	1, 39, 40
d_S	Duration of subclinical infectiousness (I_S to R; days)	$\sim gamma(\mu = 5.0, k = 4)$	Assumed to be the same duration as total infectious period for clinical cases, including preclinical transmission
	Incubation period (E to I_C; days)	$d_E + d_P$; mean 5.5 days	Derived
	Serial interval (days)	Mean ca. 7 days	Derived
u	Susceptibility to infection on contact	Calculated from R_0	Derived
y_i	Probability of clinical symptoms on infection for age group *i*	Estimated from case distributions across 6 countries	16
f	Relative infectiousness of subclinical cases	50%	Assumed
c_{ij}	Number of age-*j* individuals contacted by an age-*i* individual per day	UK-specific contact matrix	17
N_i	Number of age-*i* individuals	Demographic data	41
Δt	Time step for discrete-time simulation	0.25 days	
	Delay from onset to hospitalisation (days)	$\sim gamma(\mu = 7, k = 7)$	44, 45
	Duration of hospitalisation in non-ICU bed (days)	$\sim gamma(\mu = 8, k = 8)$	Duration based on NHS data for J12: viral pneumonia, not elsewhere classified[46].
	Duration of hospitalisation in ICU bed (days)	$\sim gamma(\mu = 10, k = 10)$	45
	Proportion of hospitalised cases that require critical care	30%	45
	Delay from onset to death (days)	$\sim gamma(\mu = 22, k = 22)$	44, 45

Table S2. Age-specific hospitalisation and fatality risk.

Based on estimates from the early COVID-19 outbreak in China.[4] The naïve CFR is obtained by dividing the number of deaths by the number of cases in a given age group, and the adjusted CFR is obtained by multiplying the naïve CFR by 0.52, following an estimation of underascertainment by Russell et al.[19]

Age group	Cases (China)	Deaths (China)	Pop. (%; China)	Naïve CFR	Adjusted CFR	Hospitalised
0–9	416	0	12.0%	0.0%	0.00%	0.0%
10–19	549	1	11.6%	0.2%	0.09%	0.8%
20–29	3619	7	13.5%	0.2%	0.10%	0.8%
30–39	7600	18	15.6%	0.2%	0.12%	1.0%
40–49	8571	38	15.6%	0.4%	0.23%	1.9%
50–59	10 008	130	15.0%	1.3%	0.68%	5.4%
60–69	8583	309	10.4%	3.6%	1.87%	15.1%
70–79	3918	312	4.7%	8.0%	4.14%	33.3%
80–89	1408	208	1.8%	14.8%	7.68%	61.8%

Table S3. Projected impact of 12-week interventions in the UK

Median and 95% prediction interval reported. Time to peak cases is measured from January 29th, 2020. Totals are calculated up to December 31, 2021.

	Base	School closures	Physical distancing	Shielding of older age groups	Self-isolation	Combination
**Total cases**	23 M (13 M–30 M)	18 M (6.2 M–26 M)	16 M (6.2 M–24 M)	17 M (6.2 M–25 M)	17 M (6.1 M–25 M)	17 M (6.5 M–26 M)
**Total deaths**	350 k (170 k–480 k)	270 k (83 k–430 k)	230 k (81 k–370 k)	210 k (73 k–350 k)	240 k (78 k–400 k)	260 k (85 k–410 k)
**Cases in peak week**	3.9 M (1.3 M–6.9 M)	2.7 M (420 k–6 M)	1.9 M (450 k–3.9 M)	2.7 M (570 k–6.1 M)	2.5 M (410 k–5.5 M)	3.1 M (620 k–5.3 M)
**Deaths in peak week**	57 k (17 k–100 k)	39 k (5.7 k–86 k)	27 k (5.8 k–58 k)	32 k (6.4 k–73 k)	34 k (5.5 k–80 k)	45 k (8 k–80 k)
**Peak ICU beds required**	200 k (61 k–370 k)	140 k (20 k–320 k)	98 k (21 k–210 k)	120 k (23 k–260 k)	120 k (19 k–290 k)	160 k (28 k–290 k)
**Peak non-ICU beds required**	390 k (110 k–700 k)	260 k (37 k–600 k)	190 k (39 k–390 k)	220 k (43 k–500 k)	230 k (36 k–560 k)	310 k (53 k–550 k)
**Time to peak cases (weeks)**	12 (9–20)	14 (10–28)	19 (12–37)	14 (10–25)	15 (10–29)	22 (17–44)

Table S4. Projected impact of control measures in the UK depending upon local versus national triggering and according to shift from centring on predicted peak.

Median and 95% prediction interval reported. Time to peak cases is measured from January 29th, 2020. Totals are calculated up to December 31, 2021.

	**Base**	**Local trigger**	**National trigger**	**Local trigger, +2 weeks**	**National trigger, +2 weeks**	**Local trigger, +4 weeks**	**National trigger, +4 weeks**	**Local trigger, +8 weeks**	**National trigger, +8 weeks**
**Total cases**	23 M (13 M–30 M)	17 M (6.9 M–25 M)	17 M (6.5 M–26 M)	16 M (5.8 M–23 M)	16 M (6.3 M–24 M)	14 M (6.2 M–21 M)	14 M (6.1 M–20 M)	15 M (4.9 M–25 M)	15 M (5 M–25 M)
**Total deaths**	350 k (170 k–480 k)	260 k (91 k–390 k)	260 k (85 k–410 k)	230 k (75 k–340 k)	230 k (83 k–340 k)	200 k (80 k–290 k)	200 k (78 k–290 k)	210 k (60 k–380 k)	200 k (61 k–380 k)
**Cases in peak week**	3.9 M (1.3 M–6.9 M)	2.8 M (610 k–4.4 M)	3.1 M (620 k–5.3 M)	1.8 M (520 k–2.7 M)	2.1 M (600 k–3.4 M)	1 M (400 k–2.9 M)	1.1 M (510 k–2.6 M)	2.6 M (320 k–6.6 M)	3 M (460 k–6.7 M)
**Deaths in peak week**	57 k (17 k–100 k)	41 k (8 k–67 k)	45 k (8 k–80 k)	26 k (6.8 k–39 k)	30 k (7.8 k–49 k)	13 k (6 k–37 k)	15 k (6.6 k–34 k)	35 k (3.9 k–90 k)	39 k (5 k–92 k)
**Peak ICU beds required**	200 k (61 k–370 k)	150 k (29 k–240 k)	160 k (28 k–290 k)	92 k (24 k–140 k)	110 k (27 k–180 k)	48 k (21 k–130 k)	54 k (23 k–120 k)	130 k (14 k–330 k)	140 k (18 k–340 k)
**Peak non-ICU beds required**	390 k (110 k–700 k)	280 k (54 k–460 k)	310 k (53 k–550 k)	170 k (46 k–260 k)	210 k (52 k–340 k)	90 k (39 k–250 k)	100 k (44 k–230 k)	240 k (26 k–630 k)	280 k (34 k–650 k)
**Time to peak cases (weeks)**	12 (9–20)	22 (16–44)	22 (17–44)	23 (13–45)	24 (13–45)	13 (10–46)	25 (10–46)	13 (9–23)	14 (9–22)

Table S5. Projected impact of control measures relating to leisure activities in the UK.

Median and 95% prediction interval reported. Time to peak cases is measured from January 29th, 2020. Totals are calculated up to September 1st, 2020.

	Background	Spectator sports banned	Leisure reduced by 75%
**Total cases**	14 M (420 k–23 M)	14 M (380 k–23 M)	12 M (47 k–22 M)
**Total deaths**	160 k (2.6 k–300 k)	160 k (2.4 k–300 k)	140 k (400–280 k)
**Cases in peak week**	1.7 M (50 k–4.6 M)	1.8 M (48 k–4.6 M)	1.2 M (2.5 k–3.7 M)
**Deaths in peak week**	20 k (340–56 k)	20 k (320–56 k)	13 k (25–44 k)
**Peak ICU beds required**	71 k (1.5 k–200 k)	71 k (1.4 k–200 k)	48 k (91–160 k)
**Peak non-ICU beds required**	130 k (2.9 k–380 k)	130 k (2.7 k–380 k)	90 k (150–300 k)
**Time to peak cases (weeks)**	16 (10–30)	16 (10–30)	17 (10–30)

Table S6. Projected impact of school closures, depending upon additional contact between children and older age groups, in the UK.

Median and 95% prediction interval given. Totals are calculated up to July 20th, 2020. “Care by elders” denotes the percentage of children under 15 for which one additional daily contact with an individual 55 years older or more is added to simulations during school closures. Time to peak cases is measured from January 29th, 2020.

	Background	School closure	School closure, 20% care by elders	School closure, 50% care by elders	School closure, 100% care by elders
**Total cases**	11 M (62 k–21 M)	7.8 M (15 k–20 M)	8 M (15 k–20 M)	8.3 M (15 k–20 M)	8.8 M (17 k–21 M)
**Total deaths**	120 k (380–270 k)	65 k (150–260 k)	67 k (160–270 k)	72 k (160–280 k)	79 k (160–290 k)
**Cases in peak week**	1.3 M (6.7 k–3.9 M)	710 k (1.4 k–3.1 M)	730 k (1.4 k–3.1 M)	760 k (1.4 k–3.2 M)	800 k (1.4 k–3.3 M)
**Deaths in peak week**	15 k (40–48 k)	7.9 k (19–39 k)	8.3 k (16–40 k)	8.8 k (19–42 k)	9.8 k (15–44 k)
**Peak ICU beds required**	53 k (190–170 k)	29 k (58–140 k)	30 k (58–140 k)	32 k (60–150 k)	35 k (56–160 k)
**Peak non-ICU beds required**	100 k (370–330 k)	54 k (90–260 k)	56 k (110–270 k)	59 k (110–280 k)	65 k (120–300 k)
**Time to peak cases (weeks)**	16 (9–24)	18 (7–23)	19 (7–23)	19 (7–23)	18 (7–23)

Table S7. Comparison of projected versus empirically-estimated reproduction numbers in the UK.

Model scenario	**Model projection for R_0**<br>Median (90% HDI)	**Empirical estimate of R**<br>Median (90% CrI)	Details of empirical estimate
**Unmitigated epidemic**	2.7 (1.6–3.5)	R_t = 2.5 (1.8–3.2)	Abbott et al.[29], 26th February 2020<br>(earliest date available at time of writing)
**Lockdown**	1.0 (0.6–1.2)	R_t = 1.0 (0.9–1.0)	Abbott et al.[29], 14th April 2020<br>(latest date available at time of writing)
		R_0 = 0.6 (95% CI: 0.4–0.9)	Jarvis et al.[30]

Table S8. Projected impact of control measures in the UK depending upon whether adherence is the same across all counties or varies from county to county.

We use the "combined" intervention with national triggering and a 4-week shift (**Table S4**) as an illustrative example. Median and 95% prediction interval reported. Time to peak cases is measured from January 29th, 2020. Totals are calculated up to December 31, 2021.

	**Base**	**Intervention, no variation**	**Intervention, with variation**
**Total cases**	23 M (13 M–30 M)	14 M (6.2 M–21 M)	14 M (6 M–21 M)
**Total deaths**	350 k (170 k–480 k)	200 k (80 k–290 k)	200 k (77 k–300 k)
**Cases in peak week**	3.9 M (1.3 M–6.9 M)	1 M (400 k–2.9 M)	950 k (390 k–3 M)
**Deaths in peak week**	57 k (17 k–100 k)	13 k (6 k–37 k)	12 k (5 k–37 k)
**Peak ICU beds required**	200 k (61 k–370 k)	48 k (21 k–130 k)	44 k (18 k–130 k)
**Peak non-ICU beds required**	390 k (110 k–700 k)	90 k (39 k–250 k)	84 k (33 k–250 k)
**Time to peak cases (weeks)**	12 (9–20)	13 (10–46)	13 (10–45)
**Total subclinical**	23 M (13 M–30 M)	14 M (6.2 M–21 M)	14 M (6 M–21 M)

Supplementary references

39 Bi Q, Wu Y, Mei S, et al. Epidemiology and Transmission of COVID-19 in Shenzhen China: Analysis of 391 cases and 1,286 of their close contacts. medRxiv 2020; 2020.03.03.20028423.

40 Nishiura H, Linton NM, Akhmetzhanov AR. Serial interval of novel coronavirus (2019-nCoV) infections. medRxiv 2020; 2020.02.03.20019497.

41 Population estimates for the UK, England and Wales, Scotland and Northern Ireland - Office for National Statistics. https://www.ons.gov.uk/peoplepopulationandcommunity/populationandmigration/populationestimates/bulletins/annualmidyearpopulationestimates/mid2018 (accessed March 29, 2020).

42 Funk S. Introduction to socialmixr. 2020; published online Jan 10. https://cran.r-project.org/web/packages/socialmixr/vignettes/introduction.html (accessed March 31, 2020).

43 Public Health England. Total UK COVID-19 Cases Update. https://www.arcgis.com/apps/opsdashboard/index.html#/f94c3c90da5b4e9f9a0b19484dd4bb14 (accessed March 30, 2020).

44 Linton NM, Kobayashi T, Yang Y, et al. Incubation Period and Other Epidemiological Characteristics of 2019 Novel Coronavirus Infections with Right Truncation: A Statistical Analysis of Publicly Available Case Data. J Clin Med Res 2020; 9. DOI:10.3390/jcm9020538.

45 Cao B, Wang Y, Wen D, et al. A Trial of Lopinavir-Ritonavir in Adults Hospitalized with Severe Covid-19. N Engl J Med 2020; published online March 18. DOI:10.1056/NEJMoa2001282.

46 NHS Digital. Hospital Admitted Patient Care Activity 2018-19. NHS Digital. https://digital.nhs.uk/data-and-information/publications/statistical/hospital-admitted-patient-care-activity/2018-19 (accessed March 29, 2020).

47 Google. COVID-19 community mobility reports. 2020. https://www.google.com/covid19/mobility/ (accessed May 6, 2020).

48 Abbott S, Hellewell J, Munday J, CMMID nCoV working group, Funk S. The transmissibility of novel Coronavirus in the early stages of the 2019-20 outbreak in Wuhan: Exploring initial point-source exposure sizes and durations using scenario analysis. Wellcome Open Research 2020; 5: 17.

49 Bedford T, Neher R, Hadfield J, Hodcroft E, Ilcisin M, Müller N. Genomic analysis of nCoV spread. Situation report 2020-01-23. 2020 https://nextstrain.org/narratives/ncov/sit-rep/2020-01-23?n=1 (accessed March 31, 2020).

50 Imai N, Cori A, Dorigatti I, et al. Report 3: Transmissibility of 2019-nCoV. https://www.imperial.ac.uk/media/imperial-college/medicine/sph/ide/gida-fellowships/Imperial-College-COVID19-transmissibility-25-01-2020.pdf (accessed March 31, 2020).

51 Majumder M, Mandl KD. Early Transmissibility Assessment of a Novel Coronavirus in Wuhan, China. SSRN Journal 2020. DOI:10.2139/ssrn.3524675.

52 Read JM, Bridgen JRE, Cummings DAT, Ho A, Jewell CP. Novel coronavirus 2019-nCoV: early estimation of epidemiological parameters and epidemic predictions. medRxiv 2020; 2020.01.23.20018549.

53 Wu JT, Leung K, Leung GM. Nowcasting and forecasting the potential domestic and international spread of the 2019-nCoV outbreak originating in Wuhan, China: a modelling study. The Lancet. 2020; 395: 689–97.

54 Zhao S, Lin Q, Ran J, et al. Preliminary estimation of the basic reproduction number of novel coronavirus

(2019-nCoV) in China, from 2019 to 2020: A data-driven analysis in the early phase of the outbreak. Int J Infect Dis 2020; 92: 214–7.

55 Liu T, Hu J, Xiao J, et al. Time-varying transmission dynamics of Novel Coronavirus Pneumonia in China. Systems Biology. 2020; 79.

Centre for Mathematical Modelling of Infectious Diseases (CMMID) COVID-19 working group

The CMMID COVID-19 working group is (members listed in random order): Thibaut Jombart, Kathleen O'Reilly, Akira Endo, Joel Hellewell, Emily S Nightingale, Billy J Quilty, Christopher I Jarvis, Timothy W Russell, Petra Klepac, Nikos I Bosse, Sebastian Funk, Sam Abbott, Graham F Medley, Hamish Gibbs, Carl A B Pearson, Stefan Flasche, Mark Jit, Samuel Clifford, Kiesha Prem, Charlie Diamond, Jon C Emery, Arminder K Deol, Simon R Procter, Kevin van Zandvoort, Yueqian Fiona Sun, James D Munday, Alicia Rosello, Megan Auzenbergs, Gwen Knight, Rein M G J Houben, Yang Liu.
